# Prolonged QT Syndrome in a 27-Year-Old Female Presenting as a Cardiac Arrest after Elective Surgery

**DOI:** 10.1155/2014/348274

**Published:** 2014-11-06

**Authors:** Aibek E. Mirrakhimov, Prakruthi Voore, Alaa M. Ali

**Affiliations:** Department of Internal Medicine, Saint Joseph Hospital, 2900 N. Lake Shore, Chicago, IL 60657, USA

## Abstract

Cardiac arrest is a true medical emergency and clinicians should base the management on American Heart Association advanced cardiac life support algorithm. The potential triggers of cardiac arrest should be sought. We present a case of a 27-year-old female who developed cardiac arrest and was later found to have congenital long QT syndrome. The patient's outcome was favorable. Discussion of the key management options will be discussed in the text.

## 1. Introduction

Cardiac arrest is a true medical emergency and clinicians should base the management on American Heart Association (AHA) advanced cardiac life support (ACLS) algorithm [1]. The potential triggers of cardiac arrest should be sought [2]. Below we present a case of a 27-year-old female who developed cardiac arrest and was later found to have congenital arrhythmogenic disorder.

## 2. Case Presentation

**Figure 1:**
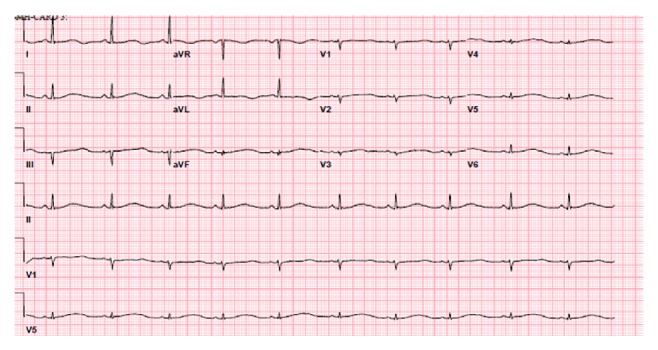
12-lead ECG showing markedly prolonged QT interval.

A 27-year-old female was admitted to the hospital for an elective laparoscopic cholecystectomy. The patient's history was significant for morbid obesity (body mass index 41.5 kg/m^2^) and cholelithiasis. The patient received midazolam and propofol for anesthesia and surgery was uneventful. The patient did not receive any anxiolytic medications prior to and after surgery. However, on the postoperative day 1 the patient suddenly became unresponsive, and code blue was called. Pulses were not palpable. Cardiopulmonary resuscitation was promptly started. Telemetry showed polymorphic ventricular tachycardia degenerating into ventricular fibrillation. The patient was cardioverted with the unsynchronized cardioversion with 120 J biphasic defibrillator device, and rapid intravenous (IV) push of magnesium was administered. Immediately after defibrillation a return of spontaneous circulation (ROSC) occurred and the patient regained consciousness. On a physical examination after the arrest her blood pressure was 110/70, respiratory rate 18, heart rate 62, temperature 97.4 F (36.3°C), and oxygen saturation 96% on room air. Lungs were clear to auscultation, and heart sounds were regular with no murmurs, rubs, or gallops, and abdominal examination was benign with prior laparoscopic incisions and extremities revealing patent pulses bilaterally and no evidence of edema. On neurological assessment, the patient was alert and oriented to place, person, and time and without focal neurological deficits. 12-lead electrocardiogram (ECG) was obtained after ROSC and is presented in Figure 1. Blood was drawn for complete blood count, chemistry panel, and serial troponins. The patient was transferred to intensive care unit (ICU) for a closer monitoring. Laboratory tests showed normal electrolytes (potassium, magnesium, and calcium) and serial ECG and troponin over 12 hours did not show evidence of acute coronary syndrome. Transthoracic echocardiogram showed ejection fraction of 70% and global normal contractility.

On a further history taking it was found that the patient was taking propranolol for some kind of cardiac condition (the patient did not remember) and that her two children (13-year-old daughter and two-month-old son) are taking propranolol too and had several episodes of cardiac arrest. However, the patient stopped taking propranolol 3 days prior to surgery for unclear reason.

12-lead ECG was remarkable for sinus rhythm, normal QRS axis, markedly prolonged QT interval (QT corrected interval of 631 milliseconds), and broad based T waves. Cardiology service was consulted. The patient received IV magnesium and propranolol was restarted. Cardiology service recommended placing of implantable cardioverter defibrillator (ICD). Patient's blood tests all came back within normal limits. The plan was to place the ICD early next morning; however, the patient arrested twice over the day due to polymorphic ventricular tachycardia associated with prolonged QT interval which is also known as torsades des pointes. Both times ROSC was achieved with the return to baseline neurological function. Eventually, ICD with properties of dual chamber pacemaker was implanted that night. The patient's further stay was uneventful, and she was discharged home on day three after ICD placement. The patient was advised to continue using propranolol. Our patient decided to undergo genetic testing and was found to have type 1 congenital long QT syndrome (was done at outside institution).

In conclusion, it is possible that both discontinuation of propranolol and stress from surgery triggered ventricular tachyarrhythmias in this patient.

## 3. Discussion

Cardiac arrest in a young patient possesses a big challenge to clinicians. Initial management of cardiac arrest should be aimed to ROSC and neurologically intact recovery. In our practice, we use AHA ACLS algorithms [1]. After patient stabilization, a search for the trigger should be performed. Common cardiac causes of sudden cardiac death include coronary artery disease, congestive heart failure, cardiomyopathies (e.g., hypertrophic cardiomyopathies), and congenital coronary artery anomalies [2]. Also, the aforementioned condition can be present in a younger patient; it is also essential to evaluate for other potential culprits such as coronary ischemia, Brugada syndrome, arrhythmogenic right ventricular dysplasia, catecholaminergic polymorphic ventricular tachycardia, and both long and short QT syndrome [2–4]. 12-lead ECG should be obtained and in many situations will provide a reliable clue to the underlying disorder. Family history should be obtained in all cases.

Long QT syndrome is a heterogeneous group of conditions that can be both congenital and acquired [5]. Long QT syndrome can be caused by electrolyte abnormalities (such as hypokalemia, hypomagnesemia, and hypercalcemia) as well as by certain medications such as fluoroquinolone antibiotics, certain antiarrhythmic medications, macrolide antibiotics, and others. A comprehensive list of medications able to prolong QT intervals can be found at https://www.crediblemeds.org/. Aforementioned electrolyte abnormalities should be aggressively corrected and culprit medications should be stopped.

Congenital long QT syndrome is a group of genetic cardiac channelopathies with 16 major subtypes with type 1 being the most common [6, 7]. Patients with congenital long QT syndrome may be completely asymptomatic or present with various ventricular arrhythmias (such as ectopy, monomorphic ventricular tachycardia, torsades de pointes, and ventricular fibrillation). Some patients may be misdiagnosed with a seizure disorder and that is why 12-lead ECG is essential in every patient with so called newly diagnosed seizure disorder to exclude prolonged QT syndrome. Cardiac arrhythmias may be triggered by physical stress, emotions, noise, or even at sleep (especially for type 3). It is important to note that QT interval should be measured at the beginning of the q waves (or if q wave is absent from the beginning of the R waves) till the end of T wave [8]. Furthermore, QT interval should be corrected for heart rate using Bazett formula. Schwartz et al. created a diagnostic algorithm based on several variables aiming to help with better identification of congenital long QT syndrome [9].

Management of clinically stable patient should include the use of certain beta blockers such as nadolol or propranolol which were shown to shorten the QT interval [9]. It is interesting to note that these beta blockers shorten the QT interval despite reducing the heart rate (slower heart rate typically prolong the QT interval). Electrolytes and in particular potassium and magnesium should be kept on the upper limit of normal. Moreover, use of medications prolonging the QT interval should be avoided if feasible. Patients with a personal or family history of cardiac arrest, recurrent unexplained syncope, congenital deafness, prolongation of QT corrected interval of 500 milliseconds or more, presence of 2 : 1 atrioventricular block, T wave electrical alternans, and type 3 of congenital long QT syndrome should be strongly considered for implantation of ICD with or without dual chamber pacemaker properties. Cardiology and electrophysiology services should be consulted to help with choice of preventive modality.
